# The Effect of ZnO, MgO, TiO_2_, and Na_2_O Modifiers on the Physical, Optical, and Radiation Shielding Properties of a TeTaNb Glass System

**DOI:** 10.3390/ma15051844

**Published:** 2022-03-01

**Authors:** Khalid I. Hussein, Mohammed S. Alqahtani, Khloud J. Alzahrani, Fawaz F. Alqahtani, Heba Y. Zahran, Ali M. Alshehri, Ibrahim. S. Yahia, Manuela. Reben, El Sayed Yousef

**Affiliations:** 1 Department of Radiological Sciences, College of Applied Medical Sciences, King Khalid University, Abha 61421, Saudi Arabia; mosalqhtani@kku.edu.sa (M.S.A.); 437808203@kku.edu.sa (K.J.A.); 2 Department of Medical Physics and Instrumentation, National Cancer Institute, University of Gezira, Wad Medani 2667, Sudan; 3 BioImaging Unit, Space Research Centre, Department of Physics and Astronomy, University of Leicester, Leicester LE1 7RH, UK; 4 Department of Radiological Sciences, College of Applied Medical Sciences, Najran University, Najran 1988, Saudi Arabia; ffalqahtani@nu.edu.sa; 5 Physics Department, Faculty of Science, King Khalid University, Abha 61413, Saudi Arabia; dr_hyzahran@yahoo.com (H.Y.Z.); amshehri@kku.edu.sa (A.M.A.); dr_isyahia@yahoo.com (I.S.Y.); 6 Research Center for Advanced Materials Science (RCAMS), King Khalid University, Abha 61413, Saudi Arabia; 7 Nanoscience Laboratory for Environmental and Bio-Medical Applications (NLEBA), Semiconductor Lab., Metallurgical Lab. 2 Physics Department, Faculty of Education, Ain Shams University, Roxy, Cairo 11757, Egypt; 8 Laboratory of Nano-Smart Materials for Science and Technology (LNSMST), Department of Physics, Faculty of Science, King Khalid University, P.O. Box 9004, Abha 61413, Saudi Arabia; 9 Faculty of Materials Science and Ceramics, AGH—University of Science and Technology, al. Mickiewicza 30, 30-059 Cracow, Poland; manuelar@agh.edu.pl

**Keywords:** metal oxide, refractive index, molar polarizability, optical energy gap, MAC, LAC, PRE

## Abstract

Novel glass samples with the composition 75TeO_2_–5Ta_2_O_5_–15Nb_2_O_5_–5x (where x = ZnO, MgO, TiO_2_, or Na_2_O) in mole percent were prepared. The physical, optical, and gamma radiation shielding properties of the glass samples were studied over a wide energy spectrum ranging between 0.015 and 20 MeV. The glasses’ UV–vis spectra were utilized to evaluate the optical energy gap and refractive index. Glass samples had a refractive index ranging from 2.2005 to 2.0967. The results showed that the sample doped with zinc oxide (ZnO) recorded the highest density (*ρ*_glass_), molar polarizability (*α_m_*), molar refraction (*Rm*), refractive index (n), and third-order nonlinear optical susceptibility (χ^3^) and the lowest optical energy gap (E_opt_) among the samples under investigation. When comparing the current glass system with various standard glass shielding materials, the prepared glass system showed superior shielding performance at energies ranging between 40 and 85 keV. These findings indicate that the prepared glass systems can be used in diagnostic X-rays, especially in dental applications.

## 1. Introduction

Due to the dramatic increase in the use of radiation in medical and industrial applications, radiation shielding issues have become an important topic among researchers [1,2,3,4,5,6].

Several computational models have been developed over the last several years to estimate radiation shielding parameters in order to study the radiation shielding effectiveness of different kinds of materials. Gerward et al. (2004) [7] developed the program WinXcom for the Windows system for the estimation of the mass attenuation coefficient (MAC) of elements, compounds, and mixtures. The calculated MAC values are based on the reported data for the mass attenuation coefficient (MAC) provided by Hubbell and Seltzer (1995) [8]. Phy-X/PSD, a user-friendly online software product for evaluating shielding and dosimetry parameters in a virtual environment for various types of materials, was recently released by Sakar et al. (2020) [9]. Recently, Khalid et al. (2021) [10] developed a computational tool for estimating and analyzing the shielding and optical parameters for all different kinds of shielding materials.

Much research has been conducted to study the effectiveness of glass as a radiation shield [1,2,3,4,5,6,7,8,9,10,11,12,13,14,15,16,17,18]. All results concluded that glass could offer excellent shielding against radiation for different applications, such as nuclear power plants, agricultural machinery, industrial processes, and medical applications [11]. Glass can offer better shielding properties with good transparency in the range of visible light compared to other shielding materials [12,13,14,15,16,17,18].

The most common network-forming constituents of glass are silicon, phosphorus, boric, and tellurium oxides. The tellurium-oxide-based glasses have excellent transparency over a wide range of wavelengths from 400 nm to 6 μm, high refractive index, good thermal stability, non-hygroscopic nature, a low melting point, and chemical durability. It is crucial to note that tellurium oxide (TeO_2_) is a unique oxide because it can only be transformed into glass by adding a suitable modifier [19,20,21]. Oxides such as ZnO, MgO, TiO_2_, and Na_2_O are the most commonly utilized materials in medical and industrial applications, making them a popular choice [20,21]. For example, adding ZnO, Na_2_O, or MgO to glasses results in decreased crystallization rates, lowered melting temperatures, and enhanced performance in the glass forming zone [22,23]. Ziad et al. [22] studied the effect of zinc oxide as a modifier in soda lime silicate glass. Their results showed that the density and the molar volume increased as the ZnO content increased, while the optical band gap decreased as the ZnO content increased. Sayyed et al. [23] studied the effects of different modifiers, including ZnO, TiO_2_, PbO, and BaO, on the optical and shielding effectiveness of boro-tellurate glasses. Their results show that the addition of heavier oxide (PbO) exhibits a more effective shielding performance. In addition to that, systems containing TiO_2_ have a greater volume density of binding energy and reduced ionic volume. The use of high density and the dual role of oxides like Ta_2_O_5_, TiO_2_, and Nb_2_O_5_ as modifiers and network formers in glass networks improves thermal stability and refractive index [24,25], making them desirable in glass shielding technology [25,26,27,28,29].

However, TeO_2_–Ta_2_O_5_–Nb_2_O_5_ oxide glasses doped with metal modifiers (ZnO, Na_2_O, MgO, TiO_2_) have not yet been studied, although they are predicted to be one of the best shielding materials. From this perspective, the primary goal of the present work is to study the effectiveness of a novel TeTaNb glass doped with different modifiers (ZnO, MgO, TiO_2_, and Na_2_O) as a shielding material. The prepared glass samples were evaluated by measuring the radiation shielding parameters such as mass attenuation coefficient (MAC), linear attenuation coefficient (LAC), half-value layer (HVL), mean free path (MFP), effective atomic number (*Z_eff_*), and effective electron number (*N_eff_*) at certain energies (59.5, 622, 1170, and 1330 keV). The measured values were compared with the calculated theoretical values at the same energies using PHY-X/PSD [9] and MIKE software [10]. The physical and optical properties of prepared glasses, such as density and transparency, were also investigated. The results of the shielding parameters were compared with other commercial shielding materials commonly used in photon and neutron applications.

## 2. Materials and Methods

Using the melt quenching technique, glasses with the mole percent composition of 75TeO_2_–5Ta_2_O_5_–15Nb_2_O_5_–5*x* (where x = ZnO, MgO, TiO_2_, or Na_2_O) were prepared from reagent-grade TeO_2_, Ta_2_O_3,_ Nb_2_O_5_, Ta_2_O_5_, ZnO, MgO, TiO_2_, and Na_2_O. The selection of the modifiers was based on the three categories defined by Dimitrov and Komatsu [30]—semi-covalent oxides (MgO), ionic or basic oxides (ZnO, TiO_2_), and very ionic or very basic oxides (Na_2_O)—in order to study the different optical characters such as the oxide ion polarizability, cation polarizability, optical basicity, O 1s binding energy, and metal (or nonmetal) binding energy of the prepared oxides. The reagents were weighed, and the mixtures were poured into a platinum crucible and melted at a temperature of 900 °C. The melt was then cast into a graphite mold. After that, the samples were transferred to an annealing furnace at 320 °C for two hours. The samples were then left for a time to cool. The densities of the prepared glass samples were measured by the Archimedes principle using toluene as the immersion fluid. The density of each sample was calculated using the following formula:

(1) $$\rho_{glass}=\frac{W_{Free}}{\left(W_{Free}-W_{Liquid}\right)}\;\;\rho_{Liquid}$$

where $\rho_{Liquid}$ is the density of the liquid (toluene), and $W_{Free}$ and $W_{Liquid}$ are the weights of the glass samples in air and toluene, respectively. 

The glass samples were prepared by cutting, grinding, and polishing. A prism spectrometer (A V-block Pulfrich refractometer PR2, Carl Zeiss, Jena, Germany) was used to measure the refractive index of the prepared samples at a wavelength of 479.98 nm using the cadmium lamp spectrum. The optical absorption spectra were measured at wavelengths from 200 to 2500 nm using a UV–VIS–NIR spectrophotometer (JASCO V-570, Tokyo, Japan). The powder X-ray diffraction (XRD) pattern of glass samples was obtained on a Siemens Kristalloflex, D500, Karlsruhe, Germany, diffractometer over a 2θ range of 5–80°, step size 0.02°.

### 2.1. Optical Properties

The parameters that characterize the optical properties of glass samples, such as the molar volume ($V_{m}$), the oxygen molar volume ($V_{o}$), and the oxygen packing density (OPD), can be calculated using the following equations [31,32]:

(2) $$V_{m}=\left(\sum^{\;}i\;x_{i}\;m_{i}/\rho_{glass}\right)$$

(3) $$V_{o}=V_{m}\cdot \left(1/x_{i}n_{i}\right)$$

(4) $$OPD=\sum^{\;}i\frac{100\cdot \rho_{glass}\cdot N_{i}}{M_{i}}$$

where $x_{i}$ is the element molar fraction, $m_{i}$ is the glassy composition molecular weight, and $n_{i}$ is the number of oxygen atoms in each oxide.

The molar refraction ($R_{m}$) and molar polarizability ($\alpha_{m}$), which depend on the glass refractive index (*n*), molar volume ($V_{m}$), and Avogadro number (*N_A_*), can be calculated using the following equations [31,32]:

(5) $$R_{m}=V_{m}\;\frac{\left(n+1\right)\left(n-1\right)}{n^{2}+2}$$

(6) $$\alpha_{m}=\left(\frac{3}{4\left(3.14\right)N_{A}V_{m}}\right)\cdot \left(\frac{n^{2}+2}{n^{2}-1}\right)$$

The metallization criterion, *M*, of prepared bulk glasses at different wavelengths is as follows [32]:

(7) $$M=\left(1-\frac{n^{2}-1}{n^{2}+2}\right)=\frac{3}{n^{2}+2}$$

The third-order nonlinear optical susceptibility, *χ*^(3)^*,* can be determined by using the linear optical susceptibility:

(8) $$\chi^{\left(3\right)}={\left[\frac{\left(n^{2}-1\right)}{12.56}\right]}^{4}\times 10^{-10}\;\;\;esu$$

### 2.2. Measurement and Theoretical Evaluation of Glass Shielding Parameters

**Figure 1:**
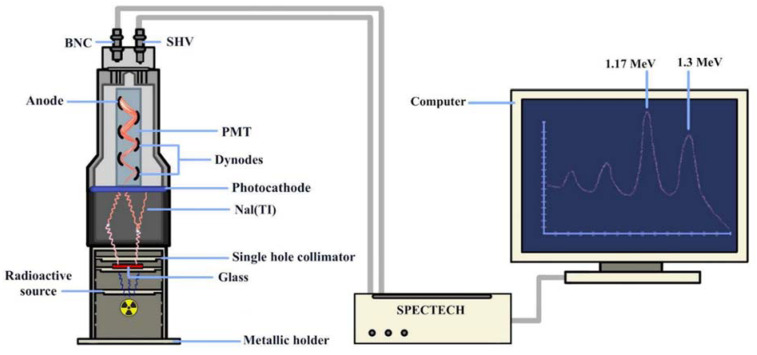
The experimental setup used for measuring the shielding parameters of the prepared samples.

A NaI detector system (SPECTECH NaI 1.5 PX 1.5/2.0 IV, S/N 010723-6, USA), connected to a computer-based multichannel analyzer (MCA), was used to measure the shielding parameters (LAC, MAC, HVL, and MFP) of the glass samples. The incident (I_0_) and transmission (I) radiation intensities of collimated beams at the detector level were measured using different gamma sources (Am^241^-5µCI-59.5 keV, Cs^137^-5µCI-662 keV, Co^60^-5µCI-1170 keV, and 1330 keV). The measured values were compared with the theoretical values at the same energies (59.5, 622, 1170, and 1330 keV) using Phy-X [9] and MIKE [10]. The shielding performance of the glass samples was evaluated and compared with that of commercially available standard materials [33]. Figure 1 shows the experimental setup used to measure the radiation shielding parameters.

The linear attenuation coefficient can be estimated from the ratio of the transmission intensity across the shielding material to the input intensity, which can be expressed mathematically by the Beer–Lambert relation [34,35]:

(9) $$\mu =\frac{ln\frac{I_{o}}{I}}{x},$$

where $I_{o}$ and I are the transmitted and input photon intensities, respectively; *x* is the thickness of the sample material. The mass attenuation coefficient (*μ_m_*) in square centimeters per gram can be estimated from the linear attenuation coefficient by dividing it by the density of the shielding material (*ρ*) [35,36,37,38].

(10) $$\mu_{m}=\frac{\mu }{\rho }$$

The theoretical mass attenuation coefficient (*μ/ρ*) of a mixture and compound can be calculated according to the following relation [35,36,37,38]:

(11) $$\frac{\mu }{\rho }=\sum_{i}w_{i}{\left(\frac{\mu }{\rho }\right)}_{i},\;$$

where $w_{i}$ is the fraction by weight of the *i*th atomic element, and ${\left(\mu /\rho \right)}_{i}$ is the mass attenuation of the *i*th atomic element. 

The probability of photon interaction with the material can be characterized by the total atom cross section ($\sigma_{a}$) and total electronic cross section ($\sigma_{e}$) using the following relations [35,36,37,38]:

(12) $$\sigma_{a}=\frac{1}{N_{A}}\sum_{i}f_{i}A_{i}{\left(\frac{\mu }{\rho }\right)}_{i},$$

(13) $$\sigma_{e}=\frac{1}{N_{A}}\sum_{j}f_{j}\frac{A_{j}}{Z_{j}}{\left(\frac{\mu }{\rho }\right)}_{j},$$

where $f_{i}$ is the fraction by mole of the *i*th atomic element, $A_{i}$ is the atomic weight of the *i*th atomic element, $Z_{j}$ is the atomic number, and $N_{A}$ is Avogadro’s constant. 

The effective atomic number is an important parameter that characterizes the properties of the shielding material in terms of photon absorption and scatter interactions. The effective atomic number, which varies with energy, can be calculated from the ratio of the atomic and electronic cross sections by the following relation [35,36,37,38].

(14) $$Z_{eff}=\frac{\sigma_{a}}{\sigma_{e}}$$

The electron density, which represents the number of electrons per unit mass of the shielding material, can be calculated using the following relation [35,36,37,38]:

(15) $$N_{e}=\frac{N_{A}}{A}Z_{eff},$$

where *A* is the mean atomic mass, equal to $\underset{i}{\sum }f_{i}A_{i}$; $f_{i}$ is the fraction by mole of the *i*th atomic element, and $A_{i}$ is the atomic weight of the *i*th atomic element.

The protection provided by the prepared glass shielding materials can be evaluated for the selected thickness by the following equation:

(16) $$RPE=\left(1-e^{-\mu_{LAC}t}\right)\;\times \;100$$

The shielding performance of glass samples was evaluated and compared with calculated values of the commercially standard glass materials RS-253, RS-360, and RS-520 [33] using MIKE [10].

## 3. Results and Discussion

The physical, optical, and radiation shielding characteristics of a prepared TeTaNb glass system doped with several modifiers (ZnO, MgO, TiO_2_, and Na_2_O) were investigated at energy levels ranging between 0.015 and 15 MeV. Table 1 shows the compositions, measured densities, and refractive indices of the glass samples under evaluation. As observed, the density decreased from 6.10235 to 5.9278 gm/cm^3^ for ZnO and Na_2_O, respectively. Incorporating ZnO into TeTaNb tellurite glasses increases the density due to the change in glass structure caused by the Zn^+2^ in breaking the Te–O network. In addition to that, the molecular weight of the modifier, the coordination numbers with interstitial spaces, and the glass crosslink density also affect the density values by changing the glass structure

### 3.1. Optical and Physical Properties

**Figure 2:**
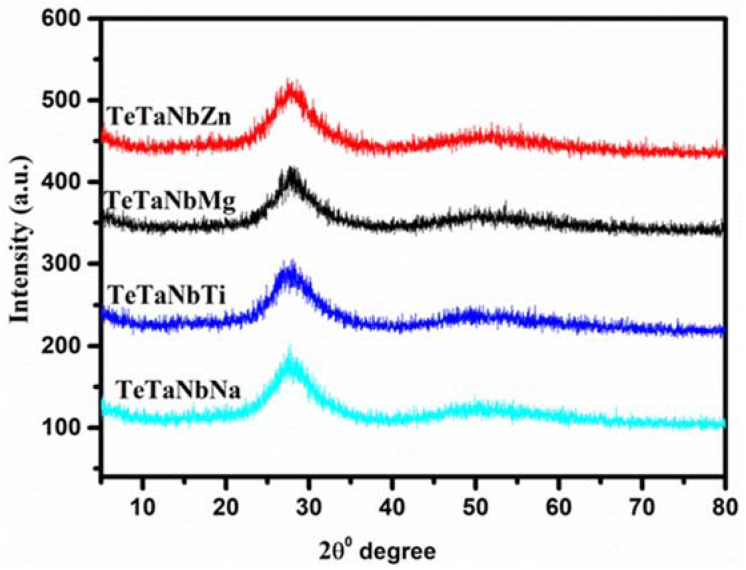
XRD chart of prepared glasses.

Figure 2 shows the X-ray diffraction (XRD) patterns of the prepared glasses. As shown in Figure 1, there are no strong peaks associated with crystalline phases. The absence of strong diffraction peaks indicates the absence of a crystalline phase, and the wide diffraction pattern confirms the amorphous nature of the prepared glasses.

Table 2 shows the calculated values of molar volume (*V_m_*), molar volume oxygen (*V_o_*), oxygen packing density (*OPD*), and energy gap. As shown in Table 2, the values of *V_m_* and *V_o_* increased from 26.08 to 26.69 m^3^/mol and from 12.722 to 13.02 m^3^/mol, respectively. The *OPD* value was reduced from 78.60 to 76.82 gm·atm·L^−1^.

The changes in the values of *V_m_*, *V_o_*, and *OPD* are influenced by a variety of parameters, such as the element molecular weight, the bond length, the number of oxygen atoms, the coordination number, and the cation radius. The unit structure of TeO_2_ glasses consists of trigonal bipyramidal TeO_4_, deformed TeO_4_, TeO_3+1_ polyhedrons, and trigonal TeO_3_ structural units. TeO_4_, TeO_3+1,_ and TeO_3_ structural units are usually labeled $Q_{4}^{4}$, $Q_{4}^{3}$, and $Q_{3}^{2}$, respectively. The subscript represents the coordination number of oxygen around the Te atom, and the superscript is the number of bridging oxygens linked to the Te atom. Therefore, an increase in the number of TeO_4_ units ($Q_{4}^{4})$ due to the influence of the added modifier leads to an increase in the sample densities in the following order: ZnO > MgO > TiO_2_ > Na_2_O. The added Ta^5+^ and Nb^5+^ with Zn^2+^ modifier fill the interstitial spaces in the tellurite matrix, thereby resulting in a denser glass [39]. Additionally, the increase in TeO_4_ concentration indicates that the glass networks are more densely packed as a consequence of increased oxygen bridging [40,41]. Table 2 and Table 3 show the optical parameter values of the prepared glasses. 

Table 2 shows the values of the Urbach energy of the prepared samples. The sample TeTaNbZn recorded the lowest energy band gap and the highest Urbach energy due to the fact that the ZnO modifier breaks the O–Te–O chains, increasing the formation of TeO_3_ and non-bridging oxygen. As reported by others [23], the formation of non-bridging oxygen (NBO) leads to the absorption band shifting to a lower energy; this causes the glass network to become less rigid. The present cations have high polarizability of the Ta^5+^ and Nb^5+^ ions and a high coordination number, creating a stable glass matrix. The addition of modifiers showed a decrease in the optical bandgap energies in the order Zn^2+^ > Mg^2+^ > Ti^2+^ > Na^2+^, attributed to fewer tightly bound oxygen anions (valence electrons). 

The TeTaNbZn glass presented the minimum value of Δ*E*, which indicates the lowest defect with a high order of BO; these results are in good agreement with findings by others [42,43].

The absorption spectra of the prepared glasses are shown in Figure 2. The optical absorption coefficient α(v) was estimated via Equation (17) [44]:

(17) $$\alpha \left(v\right)=\frac{F\left(R\right)\;}{t}=\frac{abs.}{t}\;$$

where *F*(*R*) is the Kubelka−Munk function, which can be converted to a linear absorption coefficient by applying the last equation, and t is the glass sample thickness. 

**Figure 3:**
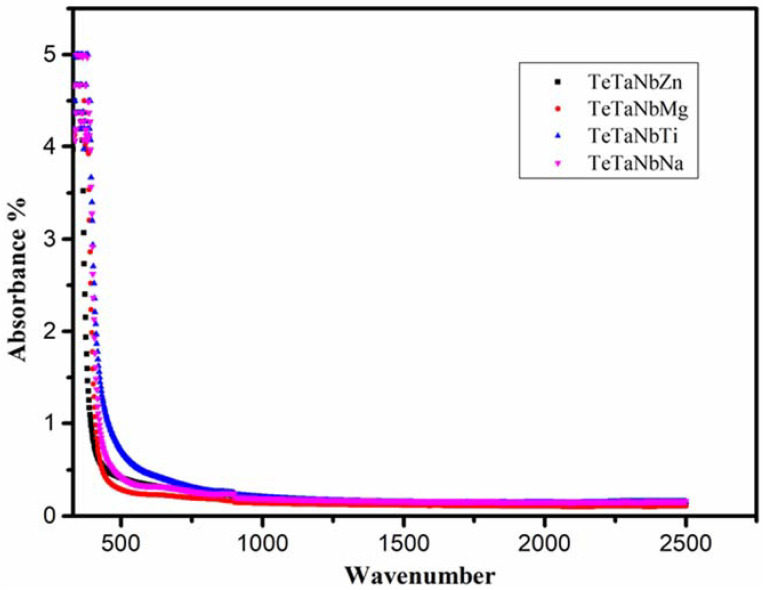
Optical absorption spectra of TeTaNb (Zn, Mg, Ti, Na) glasses.

**Figure 4:**
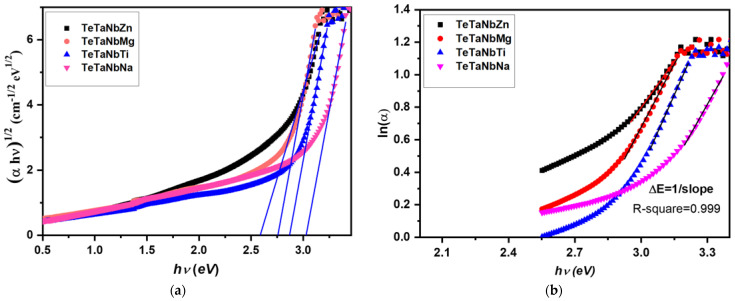
(**a**) Plot of (*αhv*)^1/2^ as a function of the photon energy (*hv*) of prepared glasses; (**b**) Plot of ln(α) as a function of the photon energy (*hv*) of prepared glasses.

Electron transitions occur when the energy of incident light exceeds the energy of the bandgap in glasses, which exhibit an energy change between valence and conduction bands ($E_{opt}$) [45]. $E_{opt}$ was calculated using the following equation [44]:

(18) $$\left(\alpha hv\right)=\frac{F\left(R\right)hv}{t}=A{\left(hv-E_{g}\right)}^{n}$$

where A is a constant, h is Plank’s constant, v is the frequency, and n is a constant that depends on the mechanism of electronic transition. For indirectly allowed transitions, n=1/ 2 can occur according to Tauc’s relations [46]. $E_{opt}$ was estimated using linear extrapolation from Tauc’s plot, which presents (*αhv*)^1/2^ vs. (*hv*), as shown in Figure 3. Table 3 presents a summary of the results. Indirect optical band gap values of 2.59, 2.75, 2.86, and 3.03 eV were recorded for TeTaNbZn, TeTaNbMg, TeTaNbTi, and TeTaNbNa, respectively. The Urbach energy (ΔE), or the localized state’s width, is used to calculate the atomic structure’s disorder degree using the following equation [47]:

(19) α(v)=βexp[hv/ΔE]

where β is a constant. The linear part of ln(α) against (hv) was used to obtain ∆*E* by taking the reciprocal. The resulting values are listed in Table 2. From Figure 4, it was found that the ΔE values ranged between 0.36 and 0.33 eV. This finding suggests that the disorder of the glass samples diminished.

Table 3 shows the molar refraction (*R_m_*) and electronic polarizability (*α_m_*), the metallization (*M*), and the linear optical susceptibility (χ^3^) of the studied glasses. The $R_{m}$ and $\alpha_{m}$ values decreased from 14.65 to 14.16 cm^3^·mol^−1^ and from 5.81 to 5.62 in Å^3^, respectively. The metallization criterion value *M* increased from 0.439 to 0.469 at 479.98 nm in the order ZnO > MgO > TiO > Na_2_O in the host glass 75TeO_2_–5Ta_2_O_5_–15Nb_2_O_5_ (TeTaNb). 

The highest value of *α_m_* (5.812 Å^3^) was recorded for TeTaNbZn, while sample TeTaNbNa recorded the lowest value of *α_m_* (5.623 Å^3^). The structure of pure TeO_2_ glass is composed of TeO_4_ trigonal bipyramids (tp) with two axial positions occupied by oxygen and two oxygens in equatorial positions. The third equatorial position is occupied by a lone pair of electrons. The network of the present glasses was modified by adding oxides, such as MgO, TiO, ZnO, or Na_2_O, leading to a combination of bridging/non-bridging oxygens, and the TeO_4_ trigonal bipyramids, which were transferred into TeO_3+1_ and TeO_3_ phases, are strongly dependent on the type of modifier added to the tellurite matrix. Tellurite with zinc oxide attributes creates β-TeO_2_, γ-TeO_2_, and the formation of Zn_2_Te_3_O_8_ units [48]. The addition of TiO_2_ to TeO_2_-based glasses obtain the chain formation of Te–O–Ti–O–Te– at lower concentration TiO_2_ whereas this chain formation becomes saturated and also creates the phase of TiO_4_ polyhedrons in the glassy matrix [49]. The addition of alkali ions Na leads to the network Te–O–Te bridges being broken and forming the non-bridging oxygens (NBO). At a low concentration of MgO content, the Te-O-Te linkage starts to break down, and TeO_4_ starts to be asymmetric with one Te-Oax bond being elongated leading to the formation of TeO_3+1_ with nonbridging oxygen (NBO) [50]. As a result, a larger number of lone pairs occupying the apex of the sp^3^ hybrid orbital in the valence shell of Te^4+^ occurred for the modifiers in the order ZnO > MgO > TiO > Na_2_O. The number of TeO_4_ units (β-TeO_2_, γ-TeO_2_) is positively correlated to the radiation protection efficiency (*RPE*); the higher the number, the better the *RPE*.

The ability to use the prepared glasses in optical applications is mostly determined by the optical refractive index (n). The value of *n* changed from 2.2005 to 2.0967, due to the variation in $\alpha_{m}$. The difference in the polarizability, $\alpha_{m}$, between the long and short Te–O bonds caused the change in the linearity values. This attribute resulted in the TeTaNbZn glass with the ZnO modifier having the highest value of *n*, with *α_m_* = 5.812 Å^3^, while sample TeTaNbNa had the lowest value of *n*, with *α_m_* = 5.623 Å^3^. It is concluded that a decrease in both values of $\alpha_{m}$ and *n* leads to an increase in the value of *M*.

The linear optical susceptibility (χ^3^) values of the prepared samples decreased from 1.49 × 10^−12^ to 1.02 × 10^−12^ esu for TeTaNbZn and TeTaNbNa, respectively; this is due to the strong dependance of χ^3^ on the values of *α_m_* and *n*.

### 3.2. Radiation Shielding Performance

**Figure 5:**
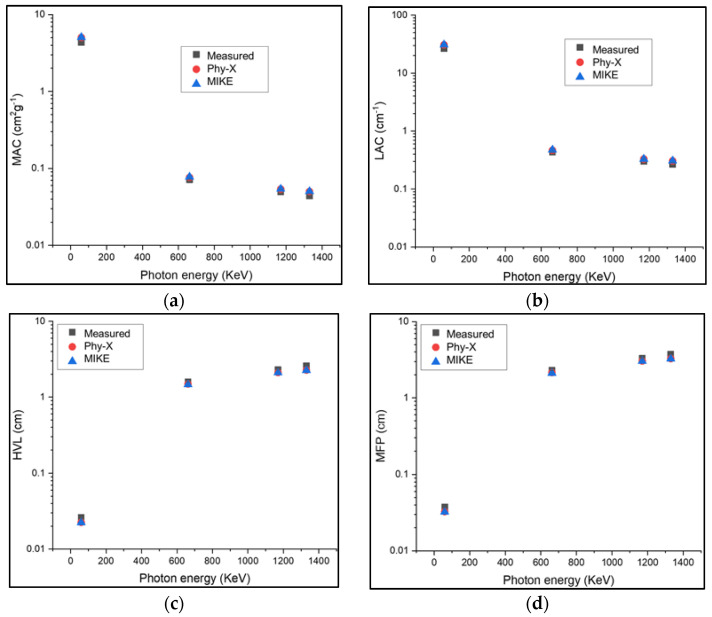
The measured and theoretical shielding parameters for TeTaNbZn glass samples at 59.5, 622, 1170, and 1330 keV: (**a**) MAC; (**b**) LAC; (**c**) HVL; (**d**) MFP.

Table 4 presents the measured MAC, LAC, HVL, and MFP values of the prepared TeTaNbZn glass samples compared to the theoretical values calculated using Phy-X and MIKE software at the specific energies of 59.5, 622, 1170, and 1330 keV. As shown in Table 4 and Figure 5, the measured values agree with the theoretical values, though they show a slight decrease.

**Figure 6:**
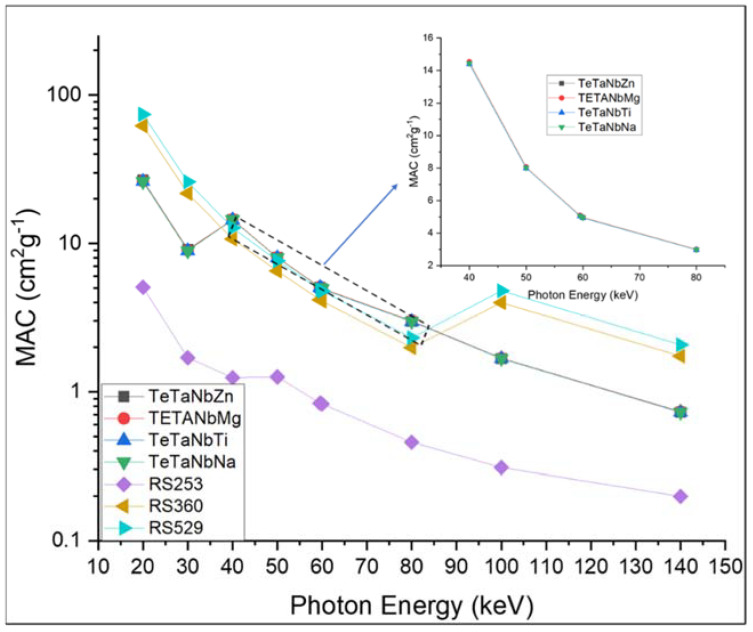
MAC values of TeTaNb (Zn, Mg, Ti, Na) glasses.

**Figure 7:**
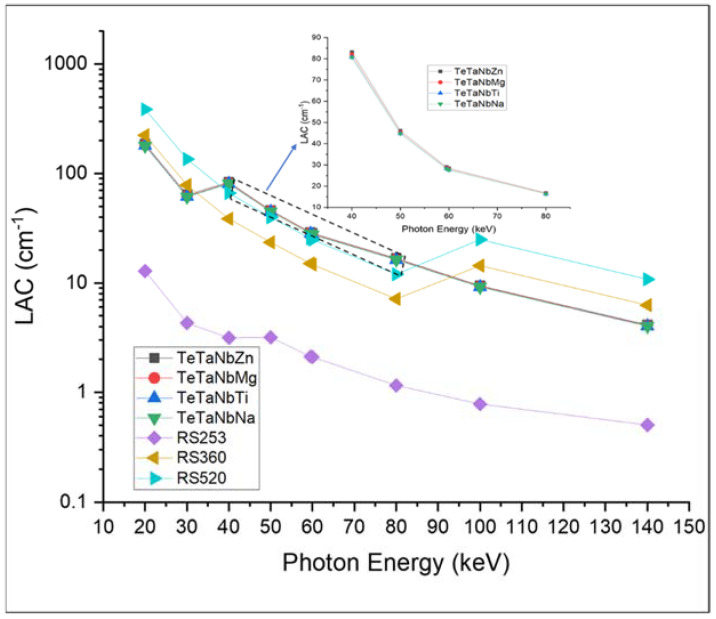
LAC values of TeTaNb (Zn, Mg, Ti, Na) glasses.

Figure 6 and Figure 7 show the mass and linear attenuation coefficients of the prepared glasses recorded at energies ranging between 15 and 150 keV. The sample doped with zinc oxide presented the highest values, while that with sodium oxide presented the lowest values. For example, the recorded LAC values at 80 keV were 18.2, 18.0, 17.70, 17.6, 1.15, 7.13, and 12.0 for TeTaNbZn, TeTaNbMg, TeTaNbTi, TeTaNbNa, RS-253, RS-360, and RS-520, respectively. The MAC and LAC of the prepared glasses are affected by the doped modifiers, and the K-absorption edges of the present modifiers are shown in Figure 6. The data show that changing the glass composition causes changes in the MAC and LAC values, demonstrating the importance of glass system composition with appropriate modifiers in terms of shielding parameters. The prepared glasses showed significant shielding efficiency in the lower energy range between 40 and 85 keV compared to the standard materials due to the low values of the K absorption edge (around 40 keV) of the modifiers under investigation, as shown in Figure 6 and Figure 7. As zinc oxide (ZnO) is the heavier oxide among the other modifiers under investigation, the addition of ZnO results in higher density, MAC, and LAC values when compared with other oxides. As illustrated in Figure 6 and Figure 7, above 85 keV photon energy, the standard materials, namely RS-360 and RS-520, recorded higher values of MAC and LAC compared to the prepared samples, which is consistent with the amount of lead oxide in these materials, as well as the effect of the lead K absorption edge at 88 keV.

**Figure 8:**
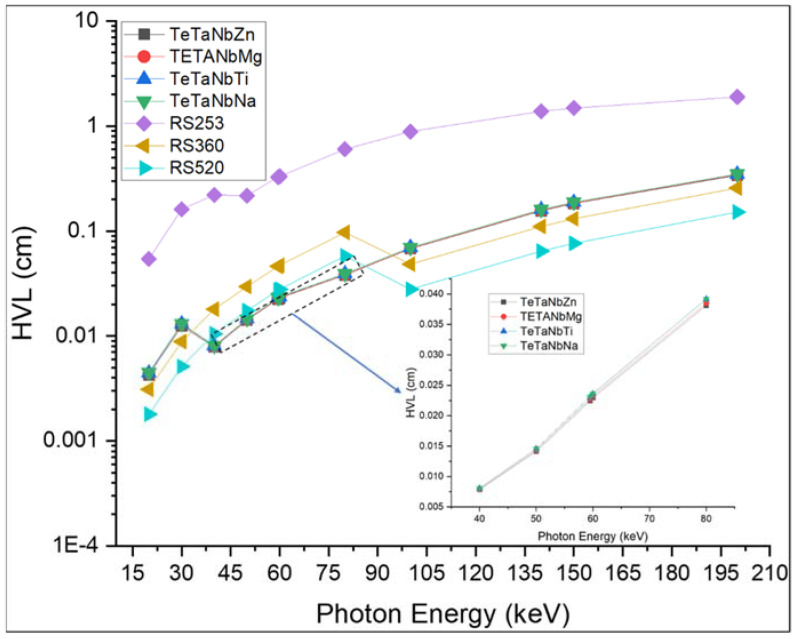
The half-value layer (HVL) of TeTaNb (Zn, Mg, Ti, Na) glasses.

**Figure 9:**
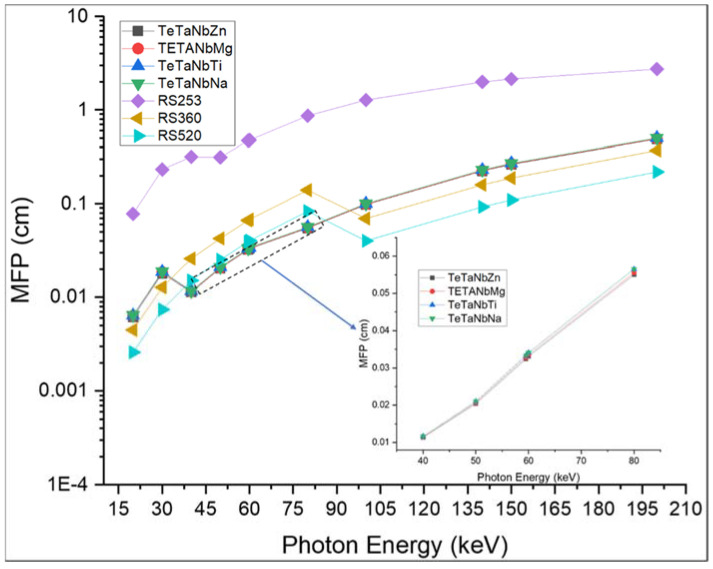
The mean free path (MFP) of TeTaNb (Zn, Mg, Ti, Na) glasses.

**Figure 10:**
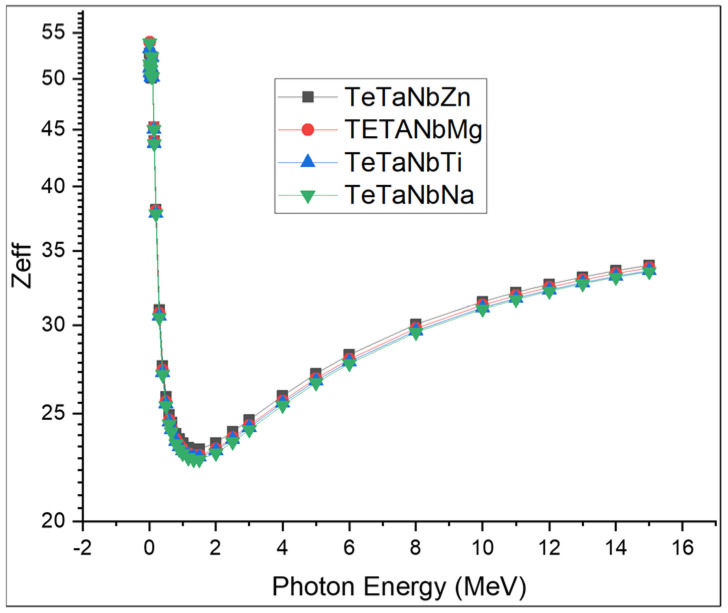
The effective atomic numbers of TeTaNb (Zn, Mg, Ti, Na) glasses.

**Figure 11:**
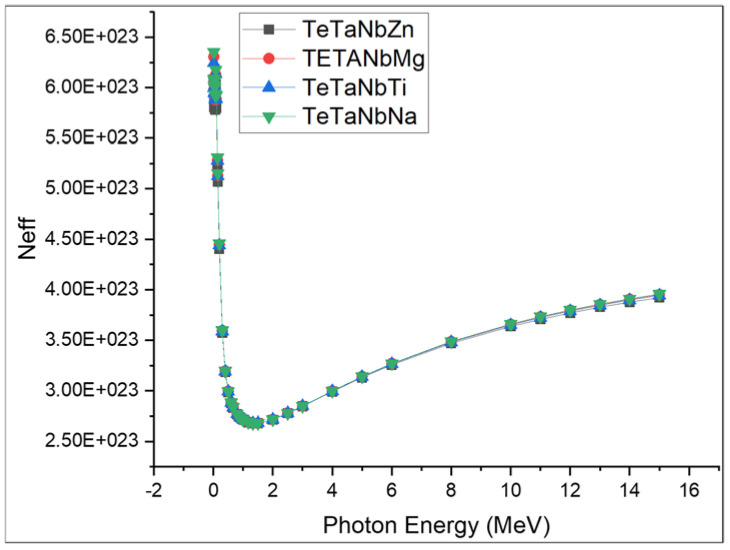
The elective electron densities (*N_eff_*) of the chosen glass systems.

Figure 8 and Figure 9 show the calculated HVL and MFP values of the prepared glass samples. The TeTaNbZn glass sample with a density of 6.1024 g/cm^3^ had the lowest HVL and MFP values, which indicate that this sample is capable of attenuating more ionizing radiation than the other glass samples under investigation. The shielding performance of the prepared glass samples was also compared to that of the commonly used glass shielding materials RS-253 G18 glass, RS-360, and RS-520. As shown in Figure 8 and Figure 9, the prepared glasses presented the lowest values in the lower energy range between 40 and 85 keV when compared with the other prepared samples and the standard commercial glass shielding materials. The high attenuation recorded is due to the photoelectric interaction process and the K-absorption edge of the samples’ constituent elements at lower energies. This effect was also observed in the values of the effective atomic numbers and effective electron numbers, as shown in Figure 10 and Figure 11. These results are consistent with the findings for both the MAC and LAC.

Figure 10 and Figure 11 show the computed values of *Z_eff_* and *N_eff_*. The TeTaNbZn glass sample presented the highest values among the investigated samples. The *Z_eff_* values for the prepared glasses were in the order TeTaNbZn > TeTaNbMg > TeTaNbTi > TeTaNbNa. Photon energy has a large influence on the *Z_eff_* and *N_eff_* values, which sharply decrease in the lower energy region due to photoelectric interactions and remain in a somewhat constant state in the medium energy region due to the Compton scattering effect, before gradually rising in the higher energy region due to the pair production domain.

**Figure 12:**
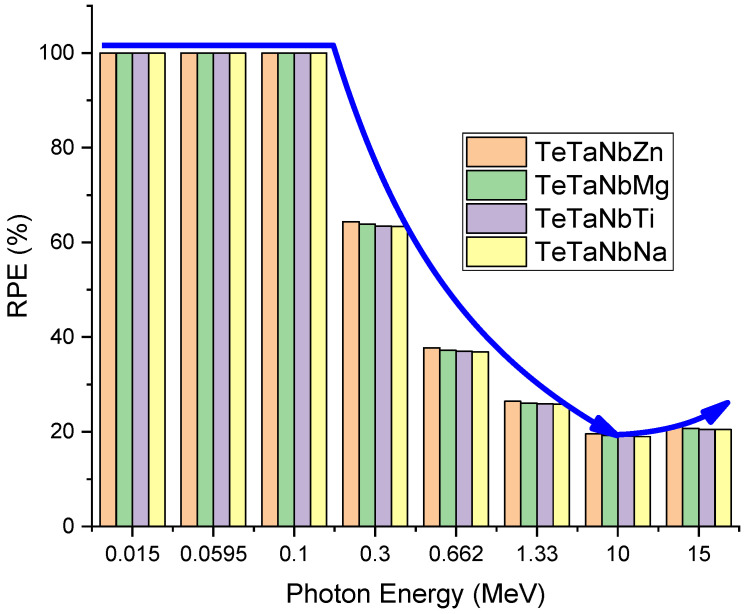
*RPE*% of prepared glasses as a function of radiation energy.

Figure 12 shows the predicted radiation protection efficiency (*RPE*) of the prepared glasses at different energy levels, ranging between 0.015 and 15 MeV using a thickness of 1 cm. At low energies up to 100 keV, the *RPE* values reached their maximum for the prepared glasses. The *RPE* percentage of the prepared glasses decreased gradually as the energy increased up to 10 MeV, then started to slowly increase for energies higher than 10 MeV. The prepared glass doped with ZnO had the highest *RPE* among the samples under investigation. These results are consistent with the findings regarding the radiation shielding parameters. The thickness of 1 cm for the present samples indicated that the present compounds have good shielding efficiency in the diagnostic range up to 100 keV; for higher energy, the thickness would need to be increased. 

The measurements and theoretical evaluation of the prepared glasses showed good shielding performance at low energies. The best shielding effectiveness in terms of MAC, LAC, HVL, MFP, and *Z_eff_* was found in the low energy range between 40 and 85 keV compared to commonly used shielding glass materials.

## 4. Conclusions

The structure and properties of a TeTaNb glass system doped with the modifiers ZnO, MgO, TiO_2_, and Na_2_O were investigated. The incorporation of ZnO into the glass matrix (TeTaNbZn) was shown to lead to a substantial increase in the density (*ρ*_glass_), molar polarizability (*α_m_*), molar refraction (*R_m_*), refractive index (n), and third-order nonlinear optical susceptibility (χ^3^) and a decrease in the optical energy gap (*E_opt_*). On the other hand, substituting ZnO by Na_2_O in the TeTaNb glass matrix led to a decrease in *ρ*_glass_, *α_m_*, n, and χ^3^ and an increase in *E_opt_*. The glass system TeTaNb doped with zinc oxide (ZnO) has the highest number of TeO_4_ units (β-TeO_2_, γ-TeO_2_), which results in a better radiation protection efficiency (*RPE*). Furthermore, the measurement and theoretical evaluation of the prepared glass showed good shielding performance at different energy ranges. The best shielding effectiveness in terms of MAC, LAC, HVL, MFP, and *Z_eff_* was found in the low energy range between 40 and 85 keV compared to standard glass shielding materials. These findings indicate that the prepared glass systems can be used in diagnostic X-ray applications, especially in dental applications. Furthermore, the radiation protection efficiency (*RPE*%) evaluation results are consistent with the previous findings regarding the MAC and LAC. Hence, the glass matrix (TeTaNb) doped with zinc oxide can be considered as a promising glass material for low energy applications in terms of transparency, thermal stability, durability, effective optical, and shielding performance.

## Figures and Tables

**Table 1:** Sample codes, compositions, densities, and refractive index values.

Sample Code	Glass Composition	Density (g·cm^−3^)	Refractive Index
TeTaNbZn	75TeO_2_–5Ta_2_O_5_–15Nb_2_O_5_–5ZnO	6.1024	2.2005
TeTaNbMg	75TeO_2_–5Ta_2_O_5_–15Nb_2_O_5_–5MgO	5.995	2.1367
TeTaNbTi	75TeO_2_–5Ta_2_O_5_–15Nb_2_O_5_–5TiO_2_	5.954	2.1186
TeTaNbNa	75TeO_2_–5Ta_2_O_5_–15Nb_2_O_5_–5Na_2_O	5.9278	2.0967

**Table 2:** The molar volume (*V_m_*), oxygen molar volume (*V_o_*), optical packing density (*OPD*), energy gap (*E_opt_*), and Urbach energy (∆*E*) of the prepared glasses.

Sample Code	*V_m_* (cm^3^·mol^−1^)	*V_o_* (cm^3^ mol^−1^)	*OPD* (mol^−1^)	Energy Gap, *E_opt_* (eV)	Urbach Energy, Δ*E* (eV)
TeTaNbZn	26.0807	12.7223	78.6023	2.59	0.36
TeTaNbMg	26.2052	12.7830	78.2286	2.75	0.31
TeTaNbTi	26.7180	12.7229	78.5986	2.86	0.49
TeTaNbNa	26.6851	13.0171	76.8218	3.03	0.34

**Table 3:** The molar refraction (*R_m_*) and electronic polarizability (*α_m_*) of the studied glasses.

Sample Code	Molar Polarizability, *α_m_*, Å^3^	Molar Refraction, *R_m_* (cm^3^ mol^−1^)	Metallization, M	χ^3^ × 10^−^^12^ Esu Third-Order Nonlinear
TeTaNbZn	5.8117	14.6455	0.4385	1.49
TeTaNbMg	5.6473	14.2312	0.4569	1.11
TeTaNbTi	5.7003	14.3647	0.4624	1.02
TeTaNbNa	5.6226	14.1690	0.4690	1.02

**Table 4:** The measured values of MAC, LAC, HVL, and MFP of the prepared glass sample TeTaNbZn compared to the theoretical values calculated using Phy-X and MIKE software.

Photon Energy (keV)	Linear Attenuation Coefficients (LAC) cm^2^/g											
	TeTaNbZn			TeTaNbMg			TeTaNbTi			TeTaNbNa		
	Exp	Phy-X	MIKE	Exp	Phy-X	MIKE	Exp	Phy-X	MIKE	Exp	Phy-X	MIKE
59.5	26.571 ± 0.05	28.982	29.035	26.273 ± 0.039	28.609	28.661	25.613 ± 0.036	28.165	28.217	25.802 ± 0.041	28.129	28.181
622	0.435 ± 0.013	0.471	0.4709	0.411 ± 0.019	0.463	0.4630	0.404 ± 0.017	0.460	0.4595	0.3903 ± 0.024	0.458	0.4576
1170	0.302 ± 0.027	0.330	0.3308	0.297 ± 0.024	0.325	0.3251	0.287 ± 0.023	0.322	0.3229	0.2800 ± 0.017	0.321	0.3214
1330	0.267 ± 0.019	0.308	0.3082	0.264 ± 0.021	0.302	0.3028	0.255 ± 0.018	0.300	0.3008	0.2489± 0.020	0.321	0.2994

## Data Availability

Not applicable.
